# Supplementary material for: Evolutionary insights into bilin biosynthesis: Functional characterization of pre‐PcyA enzymes
Source: Protein Sci. 2026 Aug 6;35(9):e70748. doi: 10.1002/pro.70748 (PMC13446030; doi:10.1002/pro.70748)
Supplement: Supplementary file 1 [file PRO-35-e70748-s001.docx]

Supporting Information

**Figure S1. Phylogenetic analysis of FDBRs.**

**Table S1. Overview of FDBRs and reaction products.**

**Figure S1. Phylogenetic analysis of FDBRs.**

A phylogeny was inferred for FDBRs as described in the Methods and is presented in simple collapsed view with the root between the CPO outgroup and all other sequences. FDBR clades examined in this study are colored, and other clades with characterized examples are in grey. Regiospecificity is indicated, and the pre-PcyA proteins examined in this study are indicated in bold.

Table S1: Overview of FDBRs and reaction products

| FDBR | Intermediate | Product |
| --- | --- | --- |
| PcyA | 18^1^,18^2^-dihydrobiliverdin | phycocyanobilin |
| PebA |  | 15,16-dihydrobiliverdin |
| PebB |  | phycoerythrobilin |
| PebS | 15,16-dihydrobiliverdin | phycoerythrobilin |
| PcyX | 15,16-dihydrobiliverdin | phycoerythrobilin |
| HY2 |  | phytochromobilin |
| PUBS | 15,16-dihydrobiliverdin | phycourobilin |
